# Assessment of the Nutritional Status of the Hemodialysis Patients by Anthropometric Measurements

**DOI:** 10.7759/cureus.18605

**Published:** 2021-10-08

**Authors:** Sajid Sultan, Kiran Nasir, Ruqaya Qureshi, Murtaza Dhrolia, Aasim Ahmad

**Affiliations:** 1 Nephrology, The Kidney Centre Post-Graduate Training Institute, Karachi, PAK

**Keywords:** anthropometric measurements, nutrition, hemodialysis, end stage renal disease, chronic kidney disease

## Abstract

Objective

This study assessed the nutritional status of end-stage renal disease (ESRD) patients on maintenance hemodialysis (MHD) by utilizing bedside anthropometric measurements.

Methods

This prospective cross-sectional study was done from November 2020 till April 2021 on ESRD patients three times a week MHD at our centre. Anthropometric measurements including body mass index (BMI), triceps skinfold thickness (TSFT), mid-arm circumference (MAC), calf circumference (CC) and handgrip strength (HGS) were measured mid-arm muscle circumference (MAMC) was calculated, and nutritional status was determined.

Results

Out of 195 patients recruited in our study, 127 (65.1%) were male. The mean age was 51.2 ± 14.8 years with a minimum of 20 and a maximum of 90 years, while the mean duration of HD was 4.6 ± 4.1 years. The majority of our patients had TSFT of 60 % to 90% 93 (47.7%), indicating mild to moderate depletion of fat stores and MAMC of >90 % 128 (65.6%), indicating good protein stores. Among all anthropometric measures, BMI was strongly associated with age (<0.001), while gender and duration of MHD were associated with TSFT (p <0.001).

Conclusion

Anthropometric measurements are easy and inexpensive bedside methods for assessing the nutritional status of ESRD patients on MHD. Our study concluded that our MHD patients have overall good nutritional status, though our young patients have low BMI and old have obesity. Male patients have weaker HGS. With the increased number of years on MHD, malnutrition increases. Our study will help to treat physicians and nutritionists for proper nutritional planning and implementation to prevent malnutrition.

## Introduction

Chronic kidney disease(CKD) is a major contributor to morbidity and mortality across the globe [1]. The incidence and prevalence of CKD are rising steadily all over the world. Along with many adverse effects on different body organs, CKD and end-stage renal disease (ESRD) are associated with body wasting and malnutrition [2].

Patients with ESRD require maintenance hemodialysis (MHD) to sustain their life. For an adult ESRD patient, the minimum dose of MHD is four hourly sessions thrice per week. In few circumstances like old age, patients with small stature and low body weight, twice per week sessions can be done. In low and middle-income countries, some centres do twice per week MHD, mainly because of the non-availability of dialysis machines and financial constraints. Patients with ESRD on MHD are subjected to emotional, financial, and physical stress. The ESRD disease burden, treatment difficulties, access to the dialysis facility, financial and socioeconomic status of the patient, dependency of the patient on their caretaker and the related logistic issues are all in themselves major contributors to the problematic life pattern and the reason for a high incidence of depression in ESRD MHD patients [3].

Protein-energy wasting (PEW) is a critical outcome of all these inter-related factors in this group of patients. According to the International Society of Renal Nutrition and Metabolism (ISRNM), PEW is a state of decreased body stores of protein and energy fuels (that is, body protein and fat masses) [4]. Once dialysis is initiated, symptoms of uremia like nausea, vomiting and decreased appetite improve with time, but many other factors leading to body wasting remain, which lead to malnutrition. Malnutrition in MHD patients is also caused by dietary factors like food restrictions, poor appetite, malabsorption and medications [5]. It also includes the pattern of diet intake and meal timing variation due to hemodialysis schedules. Nutrient losses during hemodialysis and elevated protein catabolism due to increased production of inflammatory cytokines also contribute to nutritional deficiency in hemodialysis patients [6]. Some patients may have gastrointestinal symptoms like dyspepsia, indigestion and malabsorption. These symptoms may decrease food ingestion, causing a major decrease in total calorie intake [7].

Nutritional assessment of these patients is a critical task for providing health and dietary advice. Methods of nutritional assessment include anthropometric measurements, bio-impedance, subjective global assessment (SGA) and mini nutritional assessment score (MNA) [8]. Anthropometric measurements are non-invasive techniques used to estimate total body fat, regional fat, fat and protein stores. As there is a lack of research on this subject locally, we designed this study to assess the nutritional status of ESRD patients on MHD by utilizing bedside anthropometric measurements (body mass index (BMI), triceps skinfold thickness (TSFT), mid-arm circumference (MAC), calf circumference (CC), mid-arm muscle circumference (MAMC) and handgrip strength (HGS)). This study will help in better nutritional guidance and planning for this cohort of patients.

## Materials and methods

A prospective cross-sectional study was done at The Kidney Centre Postgraduate Training Institute Karachi, Pakistan (TKC-PGTI) from November 2020 to April 2021 after approval from the hospital ethical review committee (ERC Reference No. 105-NEPH-082020). Sample selection was convenience-based sampling and the population included were adult ESRD patients on MHD thrice a week, with each session lasting 4 hours. Patients with known disabilities, especially in non-fistula arms and those on MHD twice per week, were excluded.

After getting written informed consent, demographic data [age, gender and dialysis details (duration, frequency), clinical evaluation (edema, skin changes of malnutrition)] was collected on a performed proforma. Anthropometric evaluation (height, weight, body mass index (BMI), triceps skinfold thickness (TSFT), mid-arm circumference (MAC), calf circumference (CC) and handgrip strength (HGS) were measured.

BMI was calculated by formula. BMI=Kg/m^2^. MAC and CC were measured by measuring tape, and skin-fold callipers measured TSFT. The mid-arm muscle circumference (MAMC) was calculated using the following formula: MAMC = MAC (cm)-π TSF (mm)/10. HGS of the non-fistula arm was measured using a mechanical dynamometer. For these anthropometric measurements, the patient's were seated in a sitting posture with the elbow flexed at 90 degrees and the forearm in the neutral position. Three readings were taken, and the best reading among the three was noted. According to the reference values of all these anthropometric measurements, patients were labelled as malnourished or having normal nutritional status. The BMI, MAMC and TSFT were evaluated according to the World Health Organization (WHO) [9]. The normal range of BMI is 18.5- 24.5, of MAMC in male= 25.3mm, female= 23.2mm and TSFT in male= 12.5, female= 16.5mm respectively. HGS reference ranges were taken using the predictive data of handgrip strength for the age and gender of the normal people and compared with our patient's data [12]. A TSFT below 60% of the standard value indicated severe depletion of fat reserves, 60% and 90% indicates mild to moderate depletion, and above 90% indicates adequate fat reserves. A MAMC of less than 90% indicates protein depletion; greater than 90% indicates adequate protein reserves [10]. Continuous variables like age and duration of MHD were categorized, and an association between categorical variables was established by Chi-square test using SPSS IBM version 21. A p-value of ≤ 0.05 was considered significant.

## Results

We recruited 195 patients in our study, among which 127(65.1%) were male, while 68(34.9%)were female. The mean age was 51.2 ± 14.8 years with a minimum of 20 and a maximum of 90 years, while the mean duration of HD was 4.6 ± 4.1 years. The majority of our patients had TSFT of 60 % to 90% 93(47.7%), indicating mild to moderate depletion of fat stores and MAMC of >90 % 128(65.6%), indicating good protein stores. Most of our patients had weak HGS 129(66.2%). Demographic features and anthropometric measures are described in Tables 1, 2.

When we compared the association of different age groups with nutritional status, we found that BMI was strongly associated with age (<0.001). Patients ≤ 35 years were underweight 20(100%), while patients belong to the age group of 36 to 50 years 52(48.1%) have normal BMI. All patients with grade I [11(100%)] and grade II and III obesity [8(100%)] belonged to age > 60 years (Table 3).

Gender was significantly associated with TSFT (p<0.001). Male predominantly have a higher TSFT than females. The majority of males have >90% TSFT 40(78.4%) as compared to females 11(21.6%). A similar observation was seen with MAMC, although the p-value was not significant (p=0.09). On the contrary, males have weaker HGS as compare to females [101(78.3%) versus 28(21.7%)] (Table 4).

Duration of MHD was significantly associated with TSFT (p <0.001). Most of the patients on MHD from 5 to 10 years had < 60% TSFT 17(33.3%). The duration of MHD was significantly associated with the BMI of patients (p<0.001), as we noticed that majority of underweight patients were those who were on MHD from 5 to 10 years 8 (40%) (Table 5).

**Table 1:** Demographic and anthropometric parameters of study patients n=195 TSFT- Triceps skinfold thickness; MAC- Mid arm circumference; HGS- Handgrip strength

Characteristics of patients		n (%)
Gender	Male	127(65.1)
	Female	68(34.9)
Edema on Feet		21(10.8)
Triceps skin fold thickness (TSFT)	≤ 60%	51(26.2)
	60%-90%	93(47.7)
	> 60%	51(26.2)
Mid arm circumference (MAC)	≤ 90%	67(34.4)
	>90%	128(65.6)
Hand grip strength (HGS)	Weak	129(66.2)
	Normal	66(33.8)

**Table 2:** Demographic parameters and anthropometric measurements of study patients.

Parameters of patients	Mean ± STD	Median, IQR	Minimum	Maximum
Age in years	51.2 ± 14.8	55, 21	20	90
Duration of Hemodialysis in years	4.6 ± 4.1	3, 4	3 months	22 years
Height in cm	162.5± 9.3	162.5, 11	140	187
Weight in Kg	63.4 ±13.7	63.5, 18.5	32.5	114
Body mass index (BMI)	24 ± 5	23.3, 5.9	14.8	42.2
Handgrip strength (HGS)	33.9 ± 8	34, 11	10	52
Triceps skinfold thickness (TSFT)	10.5 ± 3.8	10, 4	3	80
Mid arm circumference (MAC)	26.3 ± 3.1	26, 4	18	38
Calf circumference (CC)	29.7 ± 3	30, 4	21	39
Mid arm muscle circumference (MAMC)	23 ± 2.6	23.2, 3.4	15.5	30.3

**Table 3:** Association of different age groups with anthropometric measurements n(%).

Anthropometric measurements		≤ 35 years 37(19%)	36-50 years 52(26.7%)	51-65 years 76(39%)	> 65 years 30(15.4%)	p value
Triceps skinfold thickness (TSFT)	<60 %	12(23.5)	12(23.5)	21(41.2)	6 (11.8)	0.33
	60%-90%	19(20.4)	21(22.6)	35(37.6)	18(19.4)	
	>90%	6(11.8)	19(37.3)	20(39.2)	6(11.8)	
Mid arm muscle circumference (MAMC)	≤ 90%	19 (28.4)	15 (22.4)	23 (34.3)	10 (14.9)	0.111
	> 90 %	18 (14.1)	37 (28.9)	53 (41.4)	20 (15.6)	
Handgrip strength (HGS)	< Normal	27 (20.9)	31 (24)	53 (41.1)	18 (14)	0.439
	Normal	10 (15.2)	21 (31.8)	23 (34.8)	12 (18.2)	
Body Mass Index (BMI)	Underweight(<18)	20(100)	0	0	0	<0.001
	Normal (18.1-24.9)	17(15.7)	52(48.1)	39(36.1)	0	
	Overweight(24-29.9)	0	0	37(77.1)	11(22.9)	
	Obesity grade I (30-34.9)	0	0	0	11(100)	
	Obesity grade II&III (≥ 35)	0	0	0	8(100)	

**Table 4:** Association of gender with anthropometric measurements. n (%)

Anthropometric measurements		Male 127(65.1%)	Female 68(34.9%)	P value
Triceps skin fold thickness (TSFT)	<60 %	22 (43.1)	29 (56.9)	<0.001
	60%-90%	65(69.9)	28(30.1)	
	>90%	40(78.4)	11(21.6)	
Mid arm muscle circumference (MAMC)	≤ 90%	49 (73.1)	18 (26.9)	0.09
	> 90 %	78 (60.9)	50 (39.1)	
Handgrip strength (HGS)	< Normal	101 (78.3)	28 (21.7)	<0.001
	Normal	26 (39.4)	40 (60.6)	
Body Mass Index (BMI)	Underweight(<18)	12(60)	8(40)	0.396
	Normal (18.1-24.9)	65(60.2)	43(39.8)	
	Overweight(24-29.9)	36(75)	12(25)	
	Obesity grade I (30-34.9)	8(72.7)	3(27.3)	
	Obesity grade II&III (≥ 35)	6(75)	2(25)	

**Table 5:** Association of duration of Hemodialysis with anthropometric measurements. n (%)

Anthropometric measurements		< 1 year 22(11.3%)	1 - 3 years 82(42.1%)	3.1 - 5 years 28(14.4%)	5.1 - 10 years 44(22.6%)	> 10 years 19(9.7%)	P value
Triceps skinfold thickness (TSFT)	<60 %	5 (9.8)	9 (17.6)	5 (9.8)	17 (33.3)	15 (29.9)	<0.001
	60%-90%	13(14)	45(48.4)	17(18.3)	16(17.2)	2(2.2)	
	>90%	4(7.8)	28(54.9)	6(11.8)	11(21.6)	2(3.9)	
Mid arm muscle circumference (MAMC)	≤ 90%	8 (11.9)	25 (37.3)	13 (19.4)	13 (19.4)	8 (11.9)	0.501
	> 90 %	14 (10.9)	57 (44.5)	15 (11.7)	31 (24.2)	11 (8.6)	
Handgrip strength (HGS)	< Normal	16 (12.4)	51 (39.5)	19 (14.7)	29 (22.5)	14 (10.9)	0.824
	Normal	6 (9.1)	31 (47)	9 (13.6)	15 (22.7)	5 (7.6)	
Body Mass Index (BMI)	Underweight(<18)	4(20)	6(30)	0	8(40)	2(10)	0.002
	Normal (18.1-24.9)	13(12)	44(40.7)	19(17.6)	24(22.2)	8(7.4)	
	Overweight(24-29.9)	3(6.3)	24(50)	2(4.2)	10(20.8)	9(18.8)	
	Obesity grade I (30-34.9)	2(18.2)	5(45.5)	2(18.2)	2(18.2)	0	
	Obesity grade II&III (≥ 35)	0	3(37.5)	5(62.5)	0	0	

## Discussion

PEW is an essential concern in patients on MHD as it increases morbidity and mortality. Despite this, it is a neglected area of treatment that can be evaluated by simple bedside anthropometric measurements and easily addressed. Our study evaluated the nutritional status of MHD patients by anthropometric measurements. The mean age of the study population was 51.2 years which is comparable to similar studies done to assess nutritional status in other countries [11-12]. The mean duration of hemodialysis in our study was 4.6 ± 4.1 years.

The mean BMI of our patients is more or less similar to other studies. In a multi-centre Korean study, the mean BMI value was 23.5 ± 3.9. 13 BMI alone may be an inaccurate indicator of nutritional status among MHD patients because it does not differentiate between muscle and fat mass or provide information about body fat distribution, despite that higher BMI is found to be associated with better outcomes in MHD patients. Kim S et al. concluded that the underweight hemodialysis group with low BMI (<18.5) had a higher mortality hazard ratio as compared to the overweight group (>25.0) [13].

Edema in MHD patients is multifactorial and may be due to low albumin, excessive fluid intake, poor cardiac status, inadequate hemodialysis due to intradialytic hypotension, sepsis, poor quality of dialysis or other causes. In our study, 21 patients (10.8%) had pedal edema despite thrice per week MHD. In contrast to our study, an Indian study showed pedal edema in 50% of the study population [14]. Ideally, the anthropometric measurements should be done at the correct dry weight of the patient when he/she is free of excess fluid to give an accurate picture of the nutritional status. We did the measurements before the hemodialysis session, which could be why some patients found pedal oedema.

Mean MAMC in our study was 21±2.6 years, which is comparable to the Korean study in which mean MAMC was 24.4±2.713 and to a Pakistani study, in which mean MAMC was 20.40±4.67 [15]. Lower MAMC was found in patients with age groups less than 35 years in our study. 49 out of 127 males (38%) and 18 out of 68(26.4%) females have less than 90% MAMC in our study. Higher MAMC is predictive of a lower death hazard ratio in MHD patients [16]. While PEW, even in overweight dialysis patients, was associated with higher mortality [17].

Mean TSFT in our study was 10.5 ± 3.8. Our study showed a statically significant association of TSFT with female gender and duration of MHD. Rymarz A et al. found TSFT as a reliable method of fat mass assessment in daily routine practice in patients on MHD and patients with stage IV/V CKD compared to bio-impedance [18].

HGS is a reliable and straightforward method of evaluation of muscle function. It is routinely utilized as a function of skeletal muscle strength and function in the nutritional evaluation of the general population. The functional status of general muscle strength is correlated consistently with the extent of HGS. In an Indian study, HGS in most males was weak compared to the general population of the same age group [14]. In our study, males have weaker HGS as compared to females. HGS was measured before the dialysis session in our study, which may have affected the results. The timing (before or after the dialysis session) of the HGS assessment was relevant in a study done by Delanaye P et al. [19].

Our study has few limitations. It is a single-centre study of patients on thrice per week MHD. It did not consider the dietary routine and food consumption and did not compare this study cohort with the normal population. 

## Conclusions

Anthropometric measurements are easy and inexpensive bedside methods for the assessment of the nutritional status of MHD patients. Despite these limitations, our study was done in one of Pakistan's biggest hemodialysis units of a specialist renal centre. It will help to treat physicians to emphasize timely management of the nutritional status of MHD patients and to refer the patients to a renal nutritionist for proper nutritional planning.
